# Tuberculosis Today: Microbial Insights, Epidemiological Trends, and the Role of Molecular Diagnostics

**DOI:** 10.3390/pathogens14100965

**Published:** 2025-09-24

**Authors:** Agata Maciejak-Jastrzębska, Grażyna Sygitowicz, Sylwia Brzezińska, Kinga Bielska, Ewa Augustynowicz-Kopeć

**Affiliations:** 1 Department of Laboratory Medicine, Faculty of Pharmacy, Medical University of Warsaw, 1 Banacha Str., 02-097 Warsaw, Poland; agata.maciejak@wum.edu.pl (A.M.-J.); kingabielska@wp.pl (K.B.); 2 Department of Microbiology, National Tuberculosis and Lung Diseases Research Institute, 01-138 Warsaw, Poland; s.brzezinska@igichp.edu.pl (S.B.); e.kopec@igichp.edu.pl (E.A.-K.)

**Keywords:** tuberculosis, molecular diagnostics, NAATs, genotyping assays, MALDI-TOF MS

## Abstract

Tuberculosis (TB), caused by *Mycobacterium tuberculosis*, remains a global health problem. One of the characteristic features of mycobacteria is their exceptional resistance to environmental factors and their slow growth rate, both of which significantly prolong microbiological diagnostics. Due to the mortality rate and the rising prevalence of multidrug-resistant (MDR-TB) and extensively drug-resistant tuberculosis (XDR-TB), early detection and prompt initiation of treatment are extremely important. Traditional diagnostic methods, such as microscopic examination and culture on solid and liquid media, are still important, but are time-consuming and resource-intensive. However, the dynamic development of nucleic acid amplification techniques (NAATs), genotyping assays, and matrix-assisted laser desorption/ionization time-of-flight mass spectrometry (MALDI-TOF MS) has accelerated the identification of mycobacteria and the detection of drug resistance. Early and precise diagnosis is essential for effective disease control and improved treatment outcomes. This paper reviews the current state of knowledge on tuberculosis; including biological and structural characteristics of mycobacteria; the epidemiology of the disease; and the role of the main diagnostic methods; with a particular focus on molecular methods and MALDI-TOF MS. This paper highlights their advantages and limitations and discusses their implications for the future of TB diagnosis and control

## 1. Introduction

Tuberculosis (TB) is an infectious disease caused by acid-fast bacilli (AFB) within the *Mycobacterium tuberculosis* complex (MTBC): a group of genetically closely related mycobacteria [1,2,3]. The main members of the MTBC and their key characteristics are summarized in Table 1. Although MTBC members share over 99.95% genomic identity, they differ in host specificity, virulence, and geographic distribution (Table 1) [1,4]. Recent genomic studies have proposed the reclassification of all MTBC members as synonyms of *Mycobacterium tuberculosis* (MTB). Nevertheless, due to their biological and epidemiological relevance, they continue to be considered as distinct variants in clinical and public health contexts [4,5].

*Mycobacteria* are a widely distributed group of bacteria found in both water and soil. Several species are pathogenic to humans and animals. These are strictly aerobic, typically non-spore-forming, slightly curved rods with weak Gram staining [1,6]. Their key characteristic is acid-fastness, which results from their unique cell wall structure: mycobacteria stain with carbol fuchsin and fluorochromes, forming the basis for their microscopic identification [7].

The exceptional structure of the mycobacterial cell wall, rich in lipids (comprising approximately 60% of the dry weight), including mycolic acids, waxes, and glycolipids, imparts a hydrophobic nature and remarkable resistance to environmental stressors such as desiccation, pH fluctuations, and temperature variations [1]. This unique barrier not only impedes the penetration of harmful substances but also contributes to their intrinsic resistance to numerous antibiotics and disinfectants. Additionally, mycobacteria exhibit high tolerance to acids, alkalis, and detergents, which typically compromise the structural integrity of most bacterial cells [7,8].

A slow growth rate is characteristic of all MTBC members and, other clinically relevant slow-growing mycobacteria (e.g., *Mycobacterium avium* complex, *M. kansasii*, and *M. ulcerans*). This slow growth is attributed to prolonged cell division time of 12 to 24 h under optimal laboratory conditions. This phenomenon significantly prolongs microbiological diagnostics, as obtaining a culture result may require 2–3 weeks of incubation [1,9].

Within the MTBC, *M. tuberculosis* is the predominant etiological agent, responsible for approximately 98% of cases [10]. The primary reservoir and source of transmission are infected individuals who expel mycobacteria, particularly through coughing. Tuberculosis usually presents as pulmonary disease (80–90% of cases), though mycobacteria can also cause extrapulmonary infections affecting various organs and systems [11].

Extrapulmonary tuberculosis can involve lymph nodes, bones, joints, serous membranes, urinary system, skin, subcutaneous tissue, gastrointestinal tract, reproductive system, meninges, and brain [12]. A hallmark feature of *M. tuberculosis* is its ability to persist in the host in a latent state, wherein the bacteria remain viable but do not cause clinical symptoms (latent tuberculosis) [13].

Active pulmonary tuberculosis is characterized by a chronic cough lasting more than three weeks, initially dry but later productive, with expectoration of mucous or purulent sputum. Other symptoms may include hemoptysis, chest pain, fatigue, low-grade fever, appetite and weight loss, and night sweats [11].

In this article, we present contemporary laboratory methods used in tuberculosis diagnostics, including conventional techniques such as sputum smear microscopy and solid and liquid culture media, as well as modern approaches such as molecular diagnostics and MALDI-TOF mass spectrometry. The advantages and limitations of these methods are discussed, along with potential future directions for improving the detection and identification of *Mycobacterium tuberculosis*.

## 2. Tuberculosis—A Health Problem

Tuberculosis continues to be one of the most serious infectious diseases globally. Nearly 2 billion people are infected with MTB, with around 10% developing active TB during their lifetime [14,15]. According to the latest World Health Organization (WHO) Global Tuberculosis Report [16], in 2023, approximately 10.8 million new cases of tuberculosis were reported, representing an increase from previous years and reflecting disruption in healthcare services during the COVID-19 pandemic. Alarmingly, 1.25 million people died from the disease, including 161,000 patients co-infected with HIV, underscoring the relationship between TB and HIV infection.

Tuberculosis more commonly develops in adults (about 90% of cases), with a significantly higher incidence among men (55%). TB remains highly concentrated in low- and middle-income countries, where access to healthcare is limited. The majority of new cases (around 87% of TB cases) occur in 30 countries with the highest burden of tuberculosis, with the largest numbers of cases reported in India (26%), Indonesia (10%), China (6.8%), the Philippines (6.8%), and Pakistan (6.3%) [16].

In 2021, tuberculosis was the 10th leading cause of death worldwide. It currently remains the second leading cause of death from a single infectious agent, after COVID-19. It is also the leading cause of death among people infected with HIV and one of the most significant threats related to antimicrobial resistance, particularly due to the emergence of multidrug-resistant (MDR-TB) and extensively drug-resistant TB (XDR-TB) strains. The WHO End TB Strategy aims to reduce TB deaths by 90% and TB incidence rate by 80% by 2030 compared to 2015 levels [16].

Due to the high mortality rate and the risk of spreading drug-resistant strains, early detection of the disease, improved surveillance systems, and prompt initiation of appropriate treatment are crucial for TB control strategies worldwide.

### 2.1. Critical Analysis and Challenges in Contemporary Tuberculosis Diagnostics

Contemporary TB diagnostics, while significantly evolved, still face a range of challenges stemming from the inherent limitations of the methods employed, both conventional and molecular. Understanding these limitations is crucial for optimizing diagnostic strategies and improving treatment outcomes, especially given the global disease burden and escalating drug resistance [16].

The weakest link in TB diagnostics remains the timely identification of a patient with active disease and the collection of appropriate clinical material for testing. TB is not a condition with pathognomonic symptoms, which means that suspicion often arises too late, delaying the diagnostic process and transmission control. This diagnostic delay is particularly critical in high-burden and resource-limited settings [17].

Another major shortcoming relates to treatment monitoring: current tools do not allow for precise, early assessment of therapeutic efficacy. The limitations of existing methods are reflected in treatment success rates, which remain approximately 80% for drug-susceptible TB and fall dramatically to around 50% in MDR-TB. These gaps underline an urgent need for innovative diagnostic strategies and more reliable monitoring tools to improve patient outcomes and public health impact [18].

### 2.2. Diagnostic Tools and Capabilities

Accurate and timely TB diagnosis requires the use of modern and specific diagnostic tools that enable early detection, effective treatment, and prevention of transmission. Among these, molecular techniques, particularly nucleic acid amplification tests (NAATs), play a central role by providing rapid and precise identification of *M. tuberculosis* as well as assessment of drug susceptibility [19,20,21].

The inclusion of molecular methods in tuberculosis diagnostics should be a priority in efforts to combat the disease. However, traditional diagnostic methods, such as microscopic examination of sputum smears and bacterial cultures, are still in use and have not been completely replaced [20].

In practice, an integrated approach is most commonly used, where molecular tests are performed alongside conventional diagnostic methods, thereby significantly reducing the time needed to make a diagnosis [20,21,22,23].

### 2.3. Difficulties Related to Molecular Methods

The application of molecular techniques in the diagnosis and treatment of tuberculosis is often limited by challenges related to the storage of sputum samples and the safe transport of specimens from remote healthcare centers to central laboratories [21]. Significant difficulties persist in making an accurate diagnosis of both latent and active tuberculosis, and standardized diagnostic protocols are lacking for different forms of the disease. Additionally, access to diagnostic facilities remains a major barrier in resource-limited regions. The implementation of molecular diagnostics in such settings faces additional challenges, including expensive equipment and reagent costs, dependence on uninterrupted electricity and refrigeration, limited access to well-trained laboratory personnel, and other logistical difficulties [16,24,25].

A comprehensive diagnostic protocol requires precise drug susceptibility testing and an in-depth understanding of the genetic basis of *M. tuberculosis* drug resistance. However, complications associated with MDR-TB and the increasing prevalence of XDR-TB highlight the urgent need for improved identification techniques and enhanced drug susceptibility testing for *M. tuberculosis* [21].

### 2.4. Drug-Resistant Tuberculosis

The problem of drug-resistant tuberculosis (DR-TB) remains a serious public health threat. Particular attention is given to cases of rifampicin-resistant tuberculosis (RR-TB), as rifampicin (RIF) is the most effective first-line drug, and to MDR-TB, defined as resistance to both rifampicin and isoniazid (INH). In cases of MDR-TB or RR-TB, treatment with second-line drugs is necessary. Particularly concerning and posing significant therapeutic challenges are cases of pre-extensively drug-resistant tuberculosis (pre-XDR-TB), characterized by resistance to rifampicin, isoniazid, and a fluoroquinolone, as well as extensively drug-resistant tuberculosis, which additionally exhibits resistance to amikacin or kanamycin [26,27]. According to the 2024 WHO report [16], the number of MDR-TB/RR-TB cases worldwide has remained stable between 2020 and 2023. In 2023, 400,000 people were reported to have rifampicin-resistant or multidrug-resistant tuberculosis.

### 2.5. Vaccination Against Tuberculosis

Despite advances in vaccine research, an effective alternative to the Bacillus Calmette–Guérin (BCG) vaccine, which has been in use for over a century, has yet to be developed [16]. While widespread BCG vaccination has saved millions of lives, its efficacy in preventing pulmonary tuberculosis in adolescents and adults remains limited [11]. Recent studies have highlighted promising novel vaccine candidates, which are currently in various stages of preclinical and clinical trials. Among the most advanced are the protein subunit vaccine M72/AS01E, which entered a large Phase III efficacy trial in 2024 [28], and the live-attenuated MTBVAC, which has demonstrated favorable safety and immunogenicity in Phase 1b/2a studies [29]. According to recent WHO and TAG pipeline reports, more than a dozen additional vaccines are in development, spanning protein subunit, viral vector, and live-attenuated platforms, with the potential to provide improved and durable protection across all age groups [30,31]. Additionally, according to the WHO report [16] the rising number of drug-resistant tuberculosis cases and disruptions in healthcare services caused by the COVID-19 pandemic have significantly set back efforts to control and treat the disease. The pandemic led to delays in diagnosis and treatment interruptions, which contributed to increased transmission and poor treatment outcomes, thereby facilitating the emergence and spread of MDR-TB infections. A comprehensive strategy to combat tuberculosis requires not only improved prevention measures but also a continuous advancement of diagnostic and therapeutic tools.

## 3. Methods for Identification and Diagnosis of *M. tuberculosis* Infections

Despite significant advancements in modern diagnostic techniques, particularly molecular methods, conventional approaches remain fundamental in the identification of *M. tuberculosis* and the diagnosis of tuberculosis. The effective management of tuberculosis relies on early detection and prompt initiation of appropriate treatment to prevent disease progression and transmission. However, traditional diagnostic methods, remain labor-intensive, with prolonged turnaround times often requiring weeks to obtain definitive results.

Microscopic examination of sputum smears using Ziehl−Neelsen or auramine−rhodamine staining provides a rapid yet relatively insensitive diagnostic tool, particularly in cases with low bacterial load. Culture methods, considered the gold standard for tuberculosis diagnosis, enable not only species identification but also drug susceptibility testing. Despite their high sensitivity, these methods have major limitations, including the prolonged incubation time required for *M. tuberculosis* growth, which ranges from two to eight weeks, depending on the culture medium used [20].

To overcome these challenges NAATs have revolutionized tuberculosis detection by significantly reducing the time required for diagnosis and enabling the identification of mutations leading to drug resistance.

**Figure 1:**
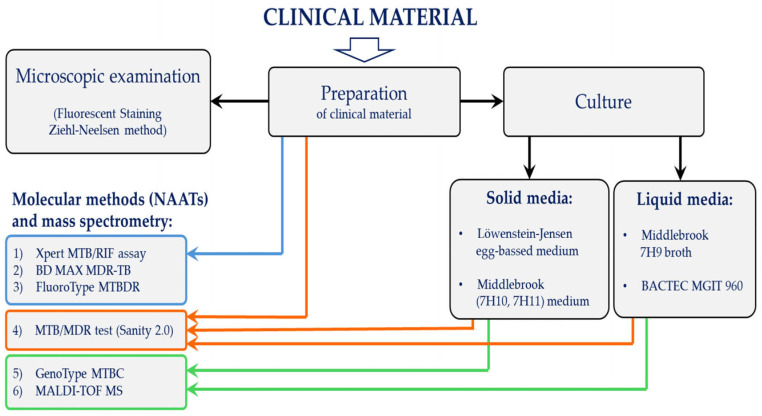
Flowchart of conventional, molecular, and mass spectrometry-based methods used for the identification and diagnosis of tuberculosis. The tests used for identification and diagnosis of tuberculosis are divided into three subgroups (marked in blue, orange, and green). Tests in the blue box—Xpert MTB/RIF assay (Cepheid, Sunnyvale, CA, USA), BD MAX MDR-TB (Becton Dickinson, Sparks, MD, USA), FluoroType MTBDR (Hain Lifescience GmbH, Nehren, Germany) are performed directly from clinical material after its processing (blue arrow). The test in the orange box—(MTB/MDR test (Xiamen Zeesan Biotech Co., Ltd., Xiamen, China) is performed directly from clinical material after processing or from liquid or solid culture (orange arrows). Tests in the green box—(GenoType MTBC (Hain Lifescience GmbH, Nehren, Germany), MALDI-TOF MS (Bruker Daltonics, Bremen, Germany) are performed from liquid or solid cultures (green arrows).

Given the complexity of tuberculosis diagnosis, an integrated workflow combining conventional methods, molecular techniques, and mass spectrometry is essential. This overview is illustrated in Figure 1 (the individual methods presented in Figure 1 will be discussed in detail in the following sections of the manuscript).

Continuous advancements in diagnostic technologies are crucial to improving the sensitivity, specificity, and accessibility of tuberculosis detection, ultimately enhancing global tuberculosis control efforts.

### 3.1. Conventional Methods for Identification of M. tuberculosis

#### 3.1.1. Microscopic Examination

##### Ziehl−Neelsen Staining of Sputum Smears

Microscopic examination of sputum smears remains the key method for identifying *M. tuberculosis*, especially in resource-limited settings. Used for over 100 years, Ziehl−Neelsen method is inexpensive, rapid, and simple, providing valuable information for confirming diagnosis and assessing infectivity. Acid-fast *M. tuberculosis* bacilli appear red, while non-acid-fast materials turn blue [24,32,33].

Despite its advantages, this method has limitations. Microscopic examination has low sensitivity (22–80%), requiring at least 10^4^–10^5^ CFU/mL of bacilli for a positive result [22,32]. In clinical practice, this means that patients with a lower bacterial load—such as those with extrapulmonary tuberculosis, early-stage disease, HIV co-infection (where dissemination might be pauci-bacillary due to immunosuppression), or in children (where obtaining an adequate sputum sample is challenging)—may receive false-negative results. Such diagnostic delays lead to disease progression, an increased risk of community transmission, and postpone the implementation of adequate treatment [33].

This technique requires careful handling of sputum samples, prompt delivery to the laboratory to prevent contamination, and proper preparation of thin smears. The accuracy of smear microscopy depends on proper specimen collection and preparation, as well as reagent quality, microscope performance, and staining time [34,35]. Importantly, microscopy cannot differentiate *M. tuberculosis* from other nontuberculous *Mycobacteria* (NTM), necessitating confirmatory tests.

#### 3.1.2. Culture on Solid and Liquid Media

Sputum culture is considered the gold standard in diagnosing active tuberculosis, with specificity > 95% and sensitivity 65−80% [36]. Culture is a much more sensitive technique than microscopic examination of sputum smears. It should be noted that even 50% of pulmonary tuberculosis cases yield negative microscopic results; therefore diagnosis is often based on culture. This method allows for reliable detection of *M. tuberculosis* in sputum at concentrations of 100−1000 CFU/mL [37,38].

A key advantage of culture is the ability to identify species and perform drug susceptibility testing, which also enables molecular epidemiology and monitoring of MDR/XDR strains [37].

Sputum culture increases the potential for diagnosing tuberculosis in the early stages of the disease. Additionally, this culture technique is used for diagnosing extrapulmonary tuberculosis, tuberculosis in children, and tuberculosis in HIV-infected individuals. Culture is also employed for assessing the effectiveness of treatment, especially in cases of previous treatment failure, evaluating drug susceptibility, and identifying specific *Mycobacterium* species [24].

However, the primary and most critical limitation of culture is the prolonged turnaround time—from 2 to 6 weeks on solid media, and even up to 10 weeks in some cases, and 7–14 days on liquid media—significantly delays treatment initiation and increases the risk of further transmission. Liquid media are more prone to contamination, which can lead to false-positive results or the need for retesting [16,37,39,40].

It is also important to note that while some diagnostic procedures involving MTBC can be performed under biosafety level 2 (BSL-2) conditions with appropriate precautions, culture and manipulation of *M. tuberculosis* require BSL-3 laboratories [24]. This is consistent with WHO biosafety guidelines recommending BSL-3 as the standard for *M. tuberculosis* culture, with BSL-2 laboratories acceptable only under very specific and strictly controlled circumstances [40].

##### Solid Media

The most commonly used solid culture media for culturing *M. tuberculosis* are Löwenstein-Jensen (LJ) egg-based medium and Middlebrook (7H10, 7H11) medium. In the case of LJ medium, the sputum sample is decontaminated, liquefied, and then spread on the surface of the medium, which is stored under controlled conditions. This method allows for the detection of *M. tuberculosis* at concentrations of 100−1000 CFU/mL, but it requires 2–6 weeks for colony development [37,38]. The Middlebrook medium provides faster growth, better colony separation, and is less prone to contamination. The 7H11 medium is preferred for culturing MDR-TB strains as it better supports the growth of isoniazid-resistant bacilli. However, a downside is its faster surface drying and the risk of formaldehyde release, which may inhibit the growth of *M. tuberculosis* [24].

##### Liquid Media

Liquid media, such as modified Middlebrook 7H9 broth, allow for faster growth of *M. tuberculosis* (7–14 days) and are used in automated system BACTEC MGIT 960. The BACTEC MGIT 960 system offers high sensitivity and a short turnaround time (10–13 days), eliminating the need for needles. Studies have shown that combining solid and liquid culture media results in better recovery of bacilli (95.5%) and faster isolation (on average 15.3 days) [39,41]. Despite faster results and higher sensitivity, liquid media are more prone to contamination and require careful prevention of cross-contamination [42].

### 3.2. Molecular Methods for the Identification of M. tuberculosis and Detection of Drug-Resistant Strains

#### 3.2.1. NAATs

Although conventional bacteriological methods for diagnosing pulmonary tuberculosis have a well-established position in clinical practice, they come with various limitations. Molecular biology techniques, particularly NAATs, have emerged as valuable alternatives [21,43]. These assays offer rapid, sensitive, specific, reliable, and reproducible results, often enabling diagnosis and treatment initiation during the same visit. This significantly reduces the risk of patient loss to follow-up and minimizes transmission [44,45].

Most NAATs target the insertion element IS6110, which is present in several species within the MTBC, allowing for the identification of *M. tuberculosis*. These tests detect ribosomal RNA or DNA of *M. tuberculosis* directly in sputum samples, regardless of smear status. NAATs have demonstrated a sensitivity of 81% in smear positive cases and 61–76% sensitivity in smear negative patients [45,46,47,48,49].

The WHO-approved molecular diagnostic tests and mass spectrometry for tuberculosis are listed in Table 2.

##### Xpert MTB/RIF Assay

The Xpert MTB/RIF test (Cepheid, Sunnyvale, CA, USA) and its latest version, Xpert MTB/RIF Ultra (Xpert Ultra; Cepheid, Sunnyvale, CA, USA), are innovative, fully automated methods based on real-time polymerase chain reaction (RT-PCR) technology. These tests represent a promising tool for the early, rapid, and specific detection of *M. tuberculosis* in sputum samples [50]. Since 2011, the WHO has recommended these tests, which enable simultaneous detection of *M. tuberculosis* infection and rifampicin resistance in individuals with symptoms of tuberculosis [51].

The principle of operation of these systems involves using a plastic cartridge (Cepheid, Sunnyvale, CA, USA) on the GeneXpert Instrument System platform (Cepheid, Sunnyvale, CA, USA). The cartridge contains all necessary reagents for DNA extraction from sputum samples, followed by amplification and detection of sequences specific to *M. tuberculosis* (IS6110 and IS1081) [21,52]. Detection of amplified products using the closed GeneXpert system is possible within 2 h, with minimal manual work and a low risk of sample contamination [53].

In addition to detecting *M. tuberculosis*, the Xpert MTB/RIF test is also used to examine the presence of mutations in the *rpoB* gene, which confer resistance to rifampicin, one of the key drugs used in tuberculosis treatment [52,54]. The WHO recommends using Xpert MTB/RIF in patients suspected of having MDR-TB and those co-infected with HIV. Recently, the WHO recommended moving away from sputum smear microscopy and instead using Xpert MTB/RIF as the first-line diagnostic test [21,51].

The analytical sensitivity of the Xpert MTB/RIF test is five genome copies of purified DNA and approximately 131 CFU/mL of *M. tuberculosis* in sputum. No significant cross-reactivity with NTM has been observed in the test [55]. The Xpert MTB/RIF test can detect pulmonary tuberculosis in 99% of cases with a positive smear result and in more than 80% of patients with a negative smear result. Furthermore, this technology can detect rifampicin resistance in *M. tuberculosis* with a sensitivity of 95.1% and specificity of 98.4% [39,52,56].

A significant achievement is the ability to use the Xpert MTB/RIF test at the point of patient care, helping reduce the risk of airborne transmission of pulmonary tuberculosis [56]. The applicability of the Xpert MTB/RIF test has also been assessed in extrapulmonary tuberculosis, including tuberculous meningitis, with promising results [57,58,59].

Moreover, the Xpert Ultra system demonstrates high sensitivity in detecting pulmonary tuberculosis in HIV-infected individuals, significantly reducing the mortality risk in this group of patients [60].

##### BD MAX MDR-TB

The BD MAX™ MDR-TB test (Becton Dickinson, Sparks, MD, USA), in conjunction with the BD MAX™ system (Becton Dickinson, Sparks, MD, USA), is an automated, qualitative molecular diagnostic test designed for the direct detection of MTBC in sputum samples from patients suspected of having pulmonary tuberculosis. In samples where MTBC DNA is detected, the BD MAX™ MDR-TB test also identifies mutations in the *rpoB* gene associated with rifampicin resistance, as well as mutations in the *katG* gene and the promoter region of *inhA*, which are linked to isoniazid resistance [61]. The BD MAX™ MDR-TB test, using the BD MAX™ system, performs sample lysis, DNA extraction, and genetic material detection Via real-time PCR. Amplification of specific DNA regions and detection of MTBC DNA and mutations associated with MDR-TB are carried out using fluorescent hybridization probes [41].

The BD MAX™ MDR-TB test offers a short turnaround time (up to 4 h), enabling rapid results and prompt therapeutic decision-making. Initial studies showed the test had a sensitivity of 93% and specificity of 97% for tuberculosis detection. It also demonstrated good sensitivity and specificity in detecting rifampicin resistance (sensitivity 90%, specificity 95%) and isoniazid resistance (sensitivity 82%, specificity 100%). These findings confirm the utility of the automated BD MAX™ MDR-TB test both in confirming pulmonary tuberculosis and identifying resistance to rifampicin and isoniazid [62].

##### FluoroType MTBDR

Rapid and precise detection of *M. tuberculosis* resistance is crucial for effective diagnosis and treatment. The FluoroType (FT) MTBDR VER 2.0 molecular test (Hain Lifescience GmbH, Nehren, Germany) provides a rapid, simple, and innovative method for identifying MTBC DNA and resistance to rifampicin and isoniazid [63,64].

The FluoroType MTBDR VER 2.0 kit (Hain Lifescience GmbH, Nehren, Germany) is based on asymmetric PCR with switch-on/switch-off probes and was analyzed using the FluoroCycler software version 1.0.1.5.5.75 (Hain Lifescience GmbH, Nehren, Germany). The FT MTBDR test amplifies target genes such as *rpoB* (for detecting mutations associated with rifampicin resistance), the *inhA* gene promoter, and the *katG* gene (for detecting mutations associated with isoniazid resistance). This test includes DNA amplification and PCR-based detection within a closed system, with automated result analysis. Single-stranded nucleic acids are identified at the end point of the reaction by analyzing the melting curve [64,65]. DNA extraction from sputum can be done manually (FluoroLyse VER 1.0, Bruker-Hain Lifescience GmbH, Nehren, Germany) or using an automated DNA extraction system (GenoXtract, Bruker-Hain Lifescience GmbH, Nehren, Germany) [66]. The FluoroType MTBDR test operates as a closed system, reducing the risk of contamination with amplicons.

Advantages of this test include a rapid turnaround time (around 3 h), reduced risk of nucleic acid contamination, and automated interpretation with data analysis options [64,67]. As a result, the FluoroType test can be a valuable tool in improving drug susceptibility testing for tuberculosis [68].

The FluoroType MTBDR test demonstrated a sensitivity of 85% and specificity of 99% in detecting *M. tuberculosis* [69]. It also showed high sensitivity (98.9%) for detecting rifampicin resistance and slightly lower sensitivity (91.7%) for detecting isoniazid resistance [64].

##### MTB/MDR Test

Sanity-2 MTB/MDR Test (Xiamen Zeesan Biotech Co., Ltd., Xiamen, China) is a fully automated molecular assay designed for the simultaneous detection of MTBC directly in clinical specimens and for the determination of resistance to two first-line anti-TB drugs: isoniazid and rifampicin [70].

This test is specifically designed for use with the Sanity 2.0 system, a fully automated platform that integrates nucleic acid extraction, RT-PCR, melting curve analysis, and result interpretation. The extraction process is carried out using the dedicated MTB DNA Extraction Kit Sanity 2.0 (Xiamen Zeesan Biotech Co., Ltd., Xiamen, China), ensuring efficient DNA purification. The DNA isolation process is based on magnetic bead technology and consists of the following steps: lysis, binding, washing, and elution, and is carried out automatically. The DNA extraction procedure is relatively simple, convenient, fast, and efficient. The Sanity-2 MTB/MDR test utilizes a TaqMan-based RT-PCR approach to detect the IS6110 sequence, which is specific to MTBC. After the RT-PCR reaction is completed, analysis of the multicolored melting curves is performed. To obtain information about the target sequence, the melting temperature (Tm) of the PCR product is calculated based on the maximum negative value of the first derivative of the fluorescence intensity as a function of temperature [70].

The drug resistance analysis includes: rifampicin resistance—detection of mutations in the *rpoB* gene (codons 507–533); isoniazid resistance—detection of mutations in the *katG* gene (codon 315), as well as mutations in the promoter regions of *ahpC* (−44 to −30 and −15 to 4) and *inhA* (−17 to −8) [70].

Preliminary comparative studies of the Sanity-2 MTB/MDR test with WHO-recommended assays demonstrated: 95% concordance in MTBC detection and 100% concordance in the detection of resistance to isoniazid and rifampicin. The MTB/MDR test demonstrated a sensitivity of 94.2% and a specificity of 97.5% in identifying rifampicin resistance, as well as slightly lower sensitivity of 84.9% and specificity of 98.0% in detecting isoniazid resistance. For MDR resistance, the test showed a sensitivity of 86.7% and a specificity of 97.7% [71].

The Sanity-2 MTB/MDR test is a rapid, automated, and highly accurate molecular assay for MTBC detection and drug resistance profiling. By combining real-time PCR with melting curve analysis, it offers a reliable solution for early identification of MDR-TB, aiding clinicians in timely treatment decisions. Its fully integrated workflow reduces the risk of contamination and human error, making it a valuable tool for TB diagnosis and resistance monitoring in clinical and public health laboratories.

##### GenoType MTBC

The GenoType MTBC test (Hain Lifescience GmbH, Nehren, Germany) is a qualitative molecular assay designed for the identification and differentiation of species within the MTBC. This assay is based on DNA-STRIP technology consisting of DNA extraction from a cultivated mycobacterial sample, multiplex amplification using biotinylated primers, and reverse hybridization of amplified DNA to membrane-bound specific probes [72].

**Figure 2:**
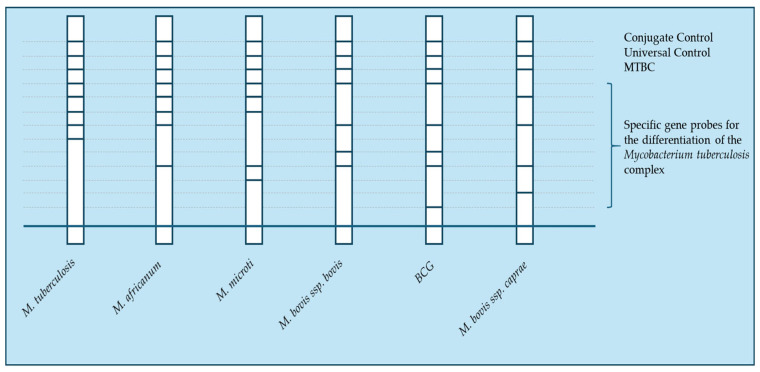
Representative result pattern of the GenoType MTBC assay. The strip shows hybridization bands for specific probes. CC (conjugate control) confirms successful conjugate binding, UC (universal control) verifies the amplification process, and MTBC control indicates the presence of MTBC DNA. The combination of band patterns allows precise differentiation of MTBC species and ensures assay reliability.

The hybridization step involves chemical denaturation of PCR products, allowing single-stranded amplicons to bind to complementary probes on the test strip. Highly specific hybridization conditions (buffer composition, temperature) ensure accurate species identification. An alkaline phosphatase-streptavidin conjugate binds to the biotin present on the amplicons. In the final step, alkaline phosphatase converts the added substrate into a dye, which appears as a colored precipitate visible on the membrane strips. The resulting pattern on the membrane strip is interpreted by comparing it with a reference pattern. To ensure quality control, each membrane strip contains two control spots that allow verification of both the test functionality and the integrity of the reagents. The presence of control bands (CC, UC, and MTBC) confirms both the validity of the reaction and the presence of MTBC genetic material (Figure 2) [72,73].

GenoType MTBC enables precise differentiation of species within the MTBC, facilitating early targeted therapy and providing valuable epidemiological insights into the potential source of infection. Comparative studies with reference methods have demonstrated an approximately 99% concordance in species identification [74]. It should be noted that the GenoType MTBC test is effective only for specific regions of the genome for which specific primers and probes have been designed.

GenoType MTBC is a rapid, highly specific, and reliable molecular assay for the identification of MTBC species, significantly enhancing TB diagnostics. By enabling precise species differentiation, it supports effective patient management, outbreak investigations, and public health interventions. The GenoType MTBC test demonstrated 100% sensitivity and 100% specificity in the detection of tuberculosis bacilli, both in liquid and solid culture media [75].

### 3.3. MALDI-TOF MS

Matrix-Assisted Laser Desorption/Ionization Time-of-Flight Mass Spectrometry (MALDI-TOF MS, Bruker Daltonics GmbH & Co. KG, Bremen, Germany) is a rapid, precise, and cost-effective method for microbial identification, particularly including *Mycobacterium* spp. The technique relies on protein profile characterization for species identification. However, its accuracy for *Mycobacterium* identification remains lower compared to other bacterial genera [76].

The high lipid content of the mycobacterial cell wall presents a major challenge in protein extraction, which is critical for reliable MALDI-TOF MS-based identification. To improve the performance, a specialized sample preparation protocol incorporating acetonitrile and formic acid treatment is required for efficient cell disruption and protein extraction [77].

MALDI-TOF MS-based identification can be performed from both solid and liquid culture media. The obtained spectra are compared against reference databases, with the MBT *Mycobacteria* IVD Library version 4.0 containing 182 *Mycobacterium* species, including four MTBC members: *M. africanum*, *M. bovis*, *M. microti*, and *M. tuberculosis*. For spectrum analysis and reliable *Mycobacterium* identification, the use of the MBT HT *Mycobacteria* IVD Module software (Bruker Daltonics GmbH & Co. KG, Bremen, Germany) is recommended [78].

The result of the analysis in the MALDI Biotyper system is presented as a score value (0.000–3.000), which indicates the likelihood of correct microbial identification based on reference spectra. A score between 0.000 and 1.699 indicates unreliable identification, scores from 1.700 to 1.999 suggest probable identification at the genus level. A score ranging from 2.000 to 2.299 indicates secure identification at the genus level and probable identification at the species level. Highly probable identification at the species level is achieved with a score between 2.300 and 3.000 [79].

A comprehensive meta-analysis of 2593 *Mycobacterium* strains reported that identification rates for MTBC species ranged from 68% for *M. bovis* to 92% for *M. tuberculosis* [80]. MALDI-TOF MS demonstrated 92.2% sensitivity and 74.1% specificity in the detection of MTB [81].

MALDI-TOF MS is a valuable tool for *Mycobacterium* identification, offering speed and cost-efficiency. However, its accuracy remains suboptimal for certain MTBC species due to protein extraction challenges. Optimization of sample preparation protocols and expansion of reference libraries are crucial for enhancing diagnostic reliability [76,78].

Limitations of the MALDI-TOF MS method include high phylogenetic similarity between microorganisms, difficulties with small and mucoid colonies, lack of results or misidentification due to the absence of a species’ protein profile in the database. To overcome these limitations, the MALDI-TOF MS method is continuously being improved, and the databases are regularly updated and expanded [82].

**Table 2:** Comparison of sensitivity, specificity, advantages, and limitations of selected molecular methods and mass spectrometry for the identification of MTBC and drug resistance.

Technology	Application	Sensitivity	Specificity	Advantages	Limitations	References
Xpert MTB/RIF	MTBC detection	99% (smear-positive)	99%	Rapid; fully automated; detects RIF resistance; minimal manual work; WHO-recommended first-line test	Limited to RIF resistance; relatively high cost; requires stable electricity and cartridges	[39,52]
		>80% (smear-negative)				
	RIF resistance identification	95%	98%			[39,56]
BD MAX MDR-TB	MTBC detection	93%	97%	Automated workflow; detects both RIF and INH resistance; shorter time to result	Limited availability; requires infrastructure and trained personnel	[62]
	RIF resistance identification	90%	95%			[62]
	INH resistance identification	82%	100%			[62]
FluoroType MTBDR	MTBC detection	85%	99%	Automated result analysis; reduced contamination risk; detects both RIF and INH resistance	Requires specific equipment; limited to first-line drugs	[69]
	RIF resistance identification	99%	100%			[64]
	INH resistance identification	92%				
MTB/MDR Test	MTBC detection	N/A	N/A	Fully automated, integrated workflow; detects RIF and broader range of INH mutations; reduced contamination risk	Limited validation studies; requires dedicated Sanity 2.0 system	N/A
	RIF resistance identification	94%	97.5%			[71]
	INH resistance identification	85%	98%			[71]
	MDR resistance	87%	98%			[71]
GenoType MTBC	MTBC detection	100%	100%	Highly accurate; differentiates MTBC species; useful in epidemiology investigations	Requires culture; no direct drug resistance detection	[75]
MALDI-TOF MS	MTBC detection	92.2%	74.1%	Rapid; cost-effective per sample; widely used in microbiology labs	Requires culture; lower accuracy for MTBC than for other bacteria; database dependent	[81]

Abbreviations: INH: Isoniazid; MTB: *Mycobacterium tuberculosis*; MTBC: *Mycobacterium tuberculosis* complex; N/A: Not applicable; RIF: Rifampicin; WHO: World Health Organization.

## 4. Summary

The diagnosis of tuberculosis, particularly in the context of drug resistance, requires the use of modern and effective techniques. Contemporary molecular methods, such as NAATs, serve as an important alternative to traditional diagnostic methods, offering rapid, precise, and reliable results.

Xpert MTB/RIF and Xpert Ultra are automated RT-PCR tests that rapidly detect *M. tuberculosis* and rifampicin resistance. Their ability to deliver results within 2 h with minimal manual work and a low risk of contamination, along with their applicability at the point of care, makes them incredibly valuable. Nevertheless, their main limitation is the narrow scope of drug resistance detection—exclusively rifampicin. In cases of suspected MDR-TB (resistance to both rifampicin and isoniazid) or XDR-TB, this test does not provide complete information, necessitating further drug susceptibility testing.

BD MAX MDR-TB and FluoroType MTBDR offer a broader detection spectrum, covering mutations associated with resistance to both rifampicin and isoniazid. While rapid (up to 4 h and around 3 h, respectively), the sensitivity for detecting isoniazid resistance is often slightly lower compared to rifampicin. They still do not cover the full spectrum of second-line drugs, which is critical in diagnosing pre-XDR-TB and XDR-TB.

The Sanity-2 MTB/MDR Test is another automated platform that detects MTBC and isoniazid and rifampicin resistance, covering a broader range of isoniazid mutations. However, further extensive validation studies are needed to fully assess its effectiveness in diverse clinical populations.

GenoType MTBC is a test for precise identification and differentiation of species within the MTBC from cultured samples. While highly accurate (approximately 99% concordance), it requires prior culture, meaning it does not reduce the time to primary diagnosis from a clinical sample. Its role is more confirmatory and epidemiological.

MALDI-TOF MS is a rapid and cost-effective method for microbial identification. However, for *Mycobacterium* spp., its accuracy remains lower compared to other bacteria (68% for *M. bovis* to 92% for *M. tuberculosis*, with overall specificity of 74.1% for MTB). This is due to the high lipid content of the mycobacterial cell wall, which complicates protein extraction and requires specialized sample preparation. Like GenoType MTBC, it requires culture of bacilli before analysis, limiting its application in rapid, primary diagnostics.

## 5. Conclusions and Future Directions

The challenges in TB diagnostics are complex, encompassing diagnostic delays (particularly with conventional methods), accessibility barriers to advanced molecular techniques, the need for rapid and comprehensive drug resistance assessment (especially in light of the growing problem of MDR-TB and XDR-TB), and difficulties in monitoring treatment effectiveness.

In light of these challenges, an integrated diagnostic approach, combining both conventional and molecular methods, is essential and forms the cornerstone of contemporary TB control strategy. Further research is needed to develop rapid, accurate, cost-effective, and readily available point-of-care testing (POCT) that can simultaneously assess drug resistance to the full spectrum of anti-tuberculosis drugs. Implementing innovative technologies, optimizing protocols, improving laboratory infrastructure, and continuous personnel training are crucial for the effective global control of tuberculosis and its drug-resistant forms.

Recent advances, such as NAATs, NGS, host biomarker research, and digital health tools, have significant potential to reshape TB diagnostics. Molecular assays already enable faster and more accurate detection of *M. tuberculosis* and drug resistance, but their integration into routine practice is still uneven due to cost, infrastructure, and accessibility barriers. Furthermore, the development of POCT, host-directed biomarkers, and AI-supported imaging holds promise for earlier detection in community settings. Crucially, advances in digital platforms may facilitate real-time monitoring of treatment adherence and outcomes, addressing one of the most critical current gaps. However, to translate these innovations into real impact, urgent research is needed on how to bridge the gap between technological progress and health system implementation, ensuring that novel diagnostic tools are affordable, scalable, and responsive to the clinical challenges of TB [83,84].

Emerging technologies such as CRISPR-based assays and nanopore sequencing represent promising next-generation approaches that could complement and, in the future, potentially replace conventional molecular diagnostics. However, their implementation in routine practice still requires extensive validation. Other innovative diagnostic methods, including LAMP, LAM detection, mNGS, and portable platforms of NAATs (e.g., Truenat), are also under evaluation, although their widespread implementation remains limited [51,85,86,87].

Advances in vaccine development represent an additional avenue for TB prevention, which, combined with innovative diagnostics, may significantly accelerate progress toward global TB elimination.

## Figures and Tables

**Table 1:** Characteristics of variants within the *Mycobacterium tuberculosis* complex (MTBC).

MTBC Variant	Characteristic Features
*M. tuberculosis*	The most common cause of tuberculosis in humans.
*M. bovis*	Responsible for zoonotic tuberculosis in humans, and tuberculosis in domestic and wild cattle.
*M. africanum*	Responsible for tuberculosis in primates in Africa. Causes pulmonary tuberculosis in humans, particularly in tropical Africa.
*M. canettii*	Causes pulmonary tuberculosis in humans, particularly in tropical Africa.
*M. caprae*	Responsible for tuberculosis in ruminants, pigs, red deer, and wild boars.
*M. pinnipedii*	Responsible for tuberculosis in seals, meerkats, and mongooses.
*M. microti*	Responsible for tuberculosis in rodents. Causes tuberculosis in immunocompromised humans.
*M. bovis BCG*	An attenuated live strain of *M. bovis* used as a vaccine for tuberculosis prevention in early childhood.
*M. mungi*	Responsible for tuberculosis in seals, meerkats, and banded mongooses.
*M. orygis*	Responsible for tuberculosis in oryx, rhinoceroses, dairy cattle, and rhesus monkeys.

## Data Availability

No new data were created or analyzed in this study.

## Abbreviations

The following abbreviations are used in this manuscript:

AFB	Acid-Fast Bacilli
BCG	Bacillus Calmette−Guérin
BD	Becton Dickinson
BSL	Biosafety Level
CFU	Colony Forming Units
COVID-19	Coronavirus Disease 2019
CRISPR	Clustered Regularly Interspaced Short Palindromic Repeats
DR-TB	Drug-Resistant Tuberculosis
HIV	Human Immunodeficiency Virus
INH	Isoniazid
LAM	Urinary lipoarabinomannan
LAMP	Isothermal amplification assays
LJ	Löwenstein–Jensen medium
MALDI-TOF MS	Matrix-Assisted Laser Desorption/Ionization Time-of-Flight Mass Spectrometry
MDR-TB	Multidrug-Resistant Tuberculosis
MGIT	Mycobacterial Growth Indicator Tube
mNGS	Metagenomic sequencing
MTB	*Mycobacterium tuberculosis*
MTBC	*Mycobacterium tuberculosis* complex
NAATs	Nucleic Acid Amplification Tests
N/A	Not applicable
NGS	Next Generation Sequencing
NTM	Nontuberculous *Mycobacteria*
PCR	Polymerase Chain Reaction
POCT	Point-of-Care Testing
pre-XDR-TB	pre-extensively drug-resistant tuberculosis
RIF	Rifampicin
RR-TB	Rifampicin-Resistant Tuberculosis
RT-PCR	Real-Time Polymerase Chain Reaction
TB	Tuberculosis
Tm	Melting Temperature
WHO	World Health Organization
XDR-TB	Extensively Drug-Resistant Tuberculosis
